# Supplementary material for: Oncolytic reprogramming of tumor microenvironment shapes CD4 T-cell memory via the IL6ra-Bcl6 axis for targeted control of glioblastoma
Source: Nat Commun. 2025 Jan 30;16:1095. doi: 10.1038/s41467-024-55455-9 (PMC11782536; doi:10.1038/s41467-024-55455-9)
Supplement: Supplementary file 1 — Supplementary Information [file 41467_2024_55455_MOESM1_ESM.pdf]

Supplementary Figure 1: related to Figure 1

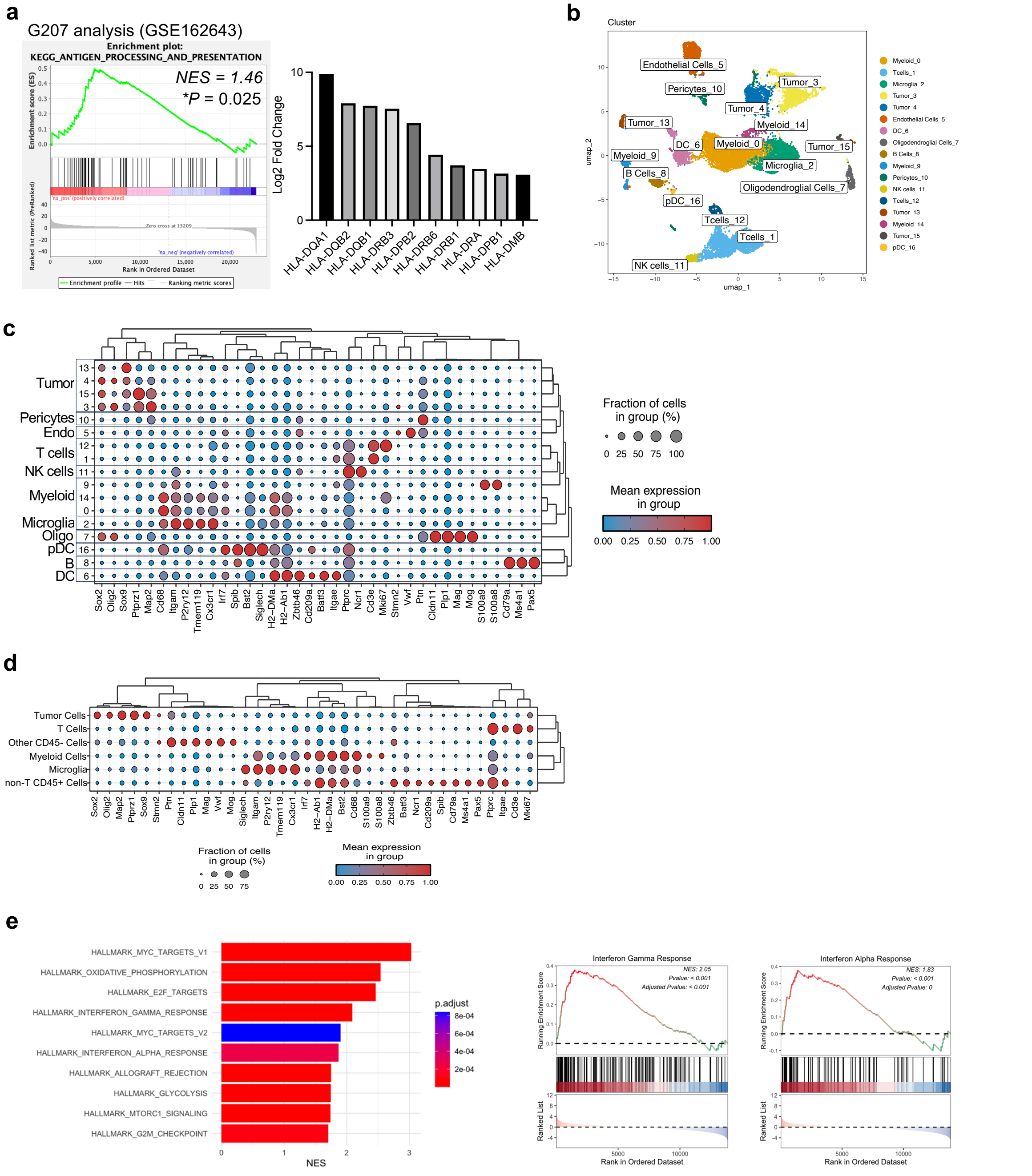

Supplementary Figure 1 (related to Figure 1): **oHSV treatment reprograms the tumor microenvironment.**

a) GSEA analysis of genes (cut off > 2 fold) upregulated in GBM patients post-G207 therapy compared to pre-therapy with survival as a covariant. KEGG antigen-processing and presentation pathway was significantly enriched (*FDR* <0.05). Right, upregulated MHCII genes.
b,c) UMAP (**b**) and dot dot (**c**) show each cell cluster identified based on mean expression of indicated marker genes, as revealed from scRNA-seq analysis in Fig. 1a.
d) Dot plot shows mean expression of indicated marker genes across each cell type after cluster grouping based on **c**. The area of the dots corresponds to the percentage fraction of cells expressing indicated genes. The color bar shows a scaled mean expression of genes in each cell cluster group.
e) Bar chart of top 10 upregulated hallmark pathways (adjusted *P* < 0.05) ranked according to Normalized Enrichment Score (NES) values (left), and GSEA analysis of indicated hallmark pathways ordered by the ranked metric of all differentially expressed genes (right) in tumor cells comparing M002-treated to saline-treated group.

Supplementary Figure 2: related to Figure 1

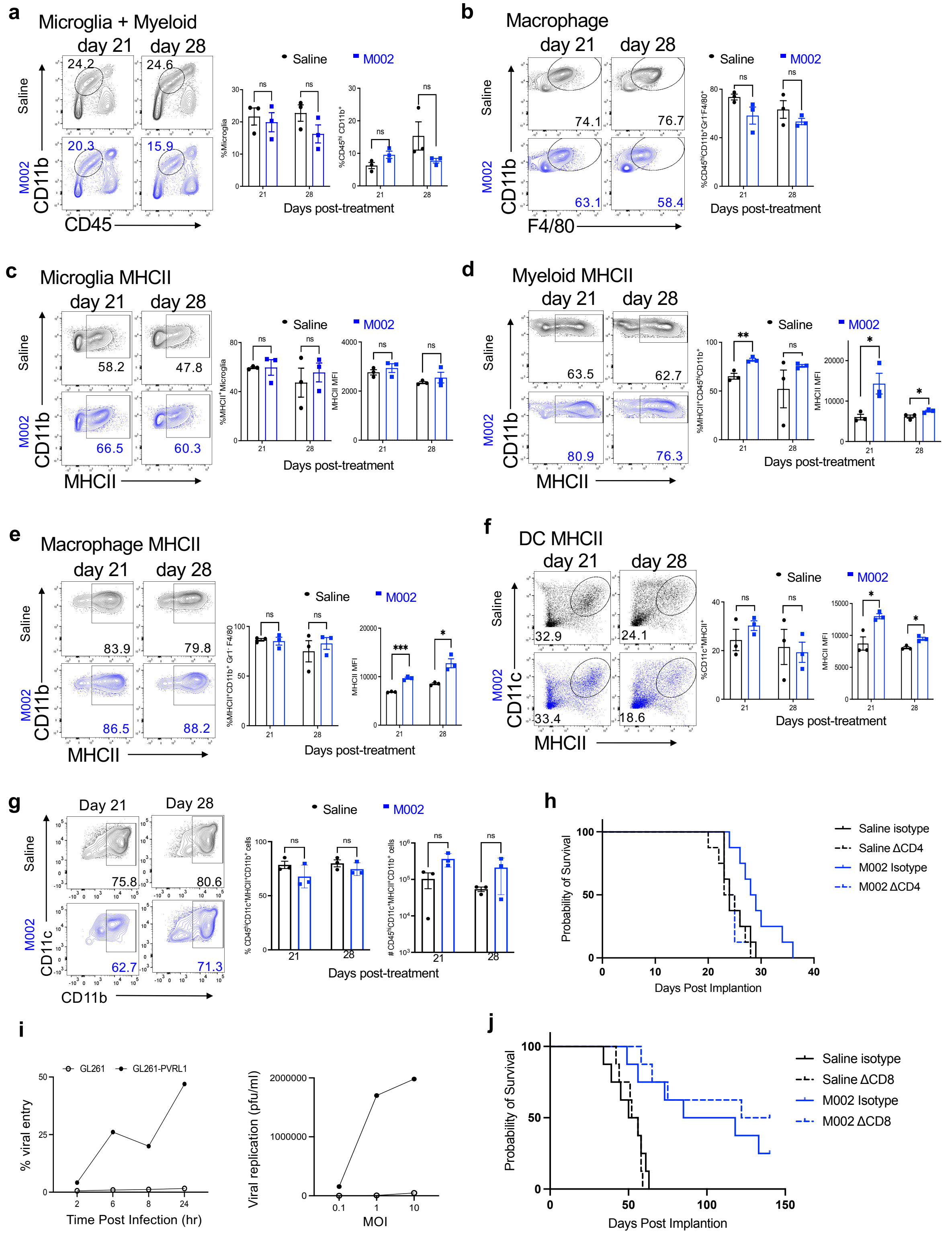

Supplementary Figure 2 (related to Figure 1): **oHSV treatment reprograms the tumor microenvironment.**

a-g) Representative plots (left) and frequency (right) of CD45^{med}CD11b^{+} microglia, CD45^{hi}CD11b^{+} myeloid cells (**a**) and CD45^{hi}CD11b^{+}Gr-1^{-}F4/80^{+} macrophage (**b**); representative plots (left), frequency (middle) of MHCII-expressing and MHCII MFI (right) on microglia (**c**), myeloid cells (**d**), macrophage (**e**), CD45^{hi}CD11c^{+}MHCII^{+} DCs (**f**), CD45^{hi}CD11c^{+}MHCII^{+} CD11b^{+} DCs (**g**) in brain tumors of mice implanted with GSC005 and treated with saline or M002 (n = 3 per group), as in Fig. 1a, at days 21 and 28 post-treatment.

h) Mice (n = 8 per group) were implanted with 100,000 GL261-PVRL1 tumor cells at day -10, and were given either M002 (blue) or saline (black) at day 0. Mice were also received either anti-CD4 (ΔCD4, dotted lines) or an isotype control antibody (Iso Ctrl, solid lines) one day before and three days after treatment. Kaplan-Meier analysis of median survival as M002 Iso Ctrl, 28.5 days; Saline Iso ctrl, 24 days; M002 ΔCD4, 24.5 days and Saline ΔCD4, 24.5 days.

i) Validating GL261-PVRL1 cells. GL261 or GL261-PVRL1 cells were infected with M201, a GFP-expressing M002 virus, at an MO1 of 5 for indicated times followed by analysis of GFP expression in cells by flow cytometry and quantification of GFP^{+} cells (left), or these cells were infected with M201 at indicated MOI for 24 hr followed by virus titers determined on Vero monolayers (right).

j) Mice (n = 8 per group) were implanted with 50,000 GSC005 tumor cells at day -11, and were given either M002 (blue) or saline (black) at day 0. Mice were also received either anti-CD8 (ΔCD8, dotted lines) or an isotype control antibody (Iso Ctrl, solid lines) one day before and three days after treatment. Kaplan-Meier analysis of median survival as M002 Iso Ctrl, 101.5 days; Saline Iso ctrl, 53 days; M002 ΔCD8, 131 days and Saline ΔCD8, 54 days, as the status on the day of resubmission.

Data represent one of two independent experiments. ns, no significance, * $P < 0.05$, ** $P < 0.01$, and *** $P < 0.001$ (a-g, unpaired two-tailed Student's t-test; h, j, log-rank test). Bars, mean ± SEM. Source data (a-j) are provided in the Source Data file.

Supplementary Figure 3: related to Figure 2

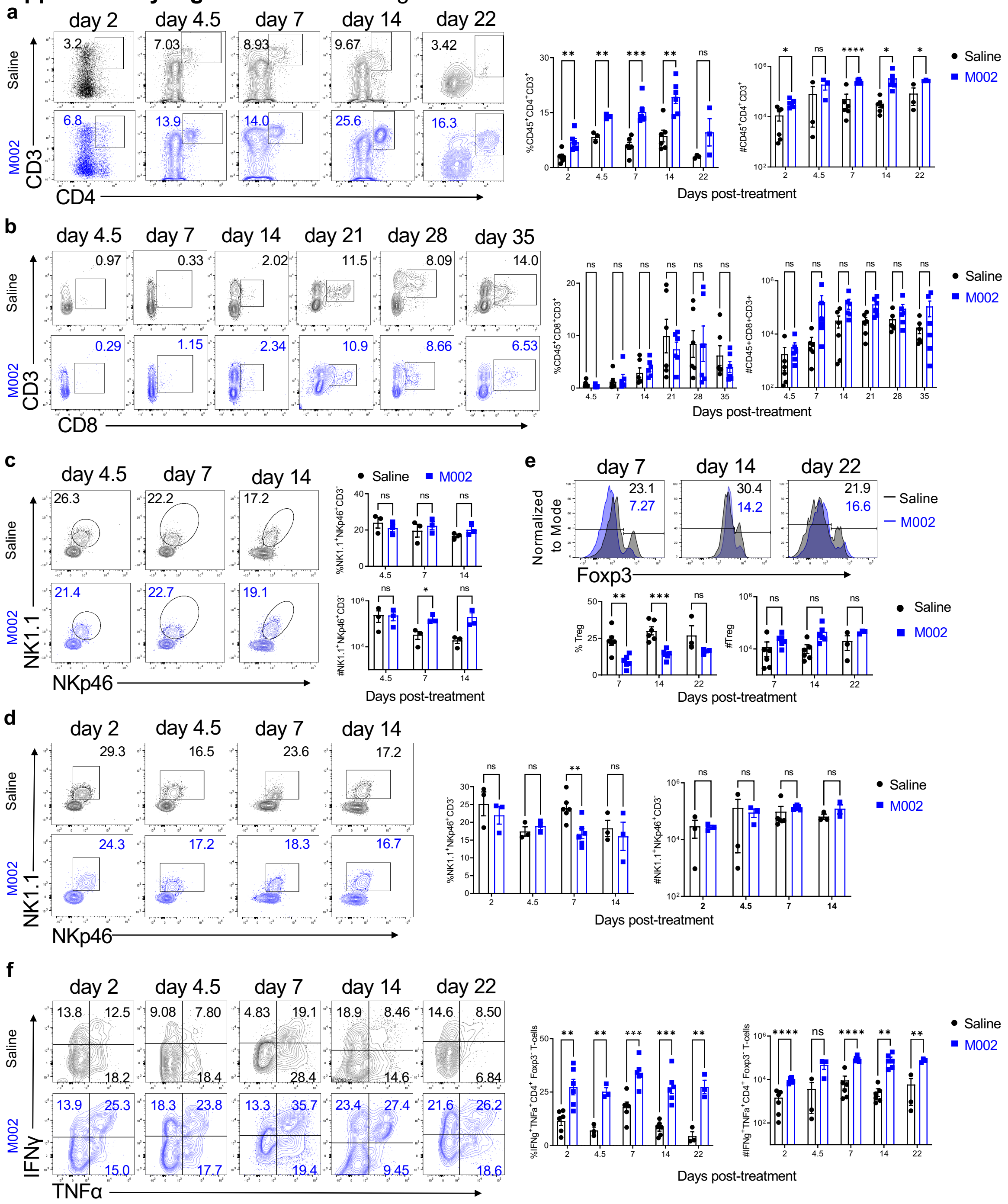

Supplementary Figure 3 (related to Figure 2): **M002 treatment expands polyfunctional effector CD4⁺ T-cells.**

a, **d-f**) Kinetic analysis of the abundance and function of CD4⁺ T-cells, and the abundance of NK and Treg cells in brain tumors of mice implanted with GL261-PVRL-1 and treated with saline or M002 10 days later. Representative plots (left), frequency (middle) and numbers (right) of $CD45^{hi}CD4^{+}CD3^{+}$ T-cells (**a**), $CD45^{hi}NK1.1^{+}NKp46^{+}CD3^{-}$ NK cells (**d**), $IFN\gamma^{+}TNF\alpha^{+}$ $CD45^{hi}CD4^{+}CD3^{+}Foxp3^{-}$ cells (**f**), and representative plots (upper), frequency (bottom left) and numbers (bottom right) of $CD45^{hi}CD4^{+}CD3^{+}Foxp3^{+}$ Treg cells (**e**). In **a**, **f**, n = 3 per group for day 4.5 and day 22; n = 6 per group for day 2, day 7 and day 14. In **d**, n = 3 per group for day 2, day 4.5 and day 14; n = 6 per group for day 7.

b, c) Kinetic analysis of the abundance of $CD8^+$ T-cells and NK cells in brain tumors of mice implanted with GSC005 and treated with saline or M002 11 days later, as in Fig. 1a. **b**) Representative plots (left), frequency (middle) and numbers (right) of $CD45^{hi}CD8^+CD3^+$ T-cells (n = 6 per group). **c**) Representative plots (left), frequency (right upper) and numbers (right bottom) of NK cells (n = 3 per group).

Each dot represents an individual mouse. Data represent one of two independent experiments (a, d-f) or are pooled from two independent experiments (b-c). ns, no significance, * $P < 0.05$, ** $P < 0.01$, *** $P < 0.001$ and **** $P < 0.0001$ (a-f, unpaired two-tailed Student's t-test). Bars, mean ± SEM. Source data (a-f) are provided in the Source Data file.

Supplementary Figure 4: related to Figure 2

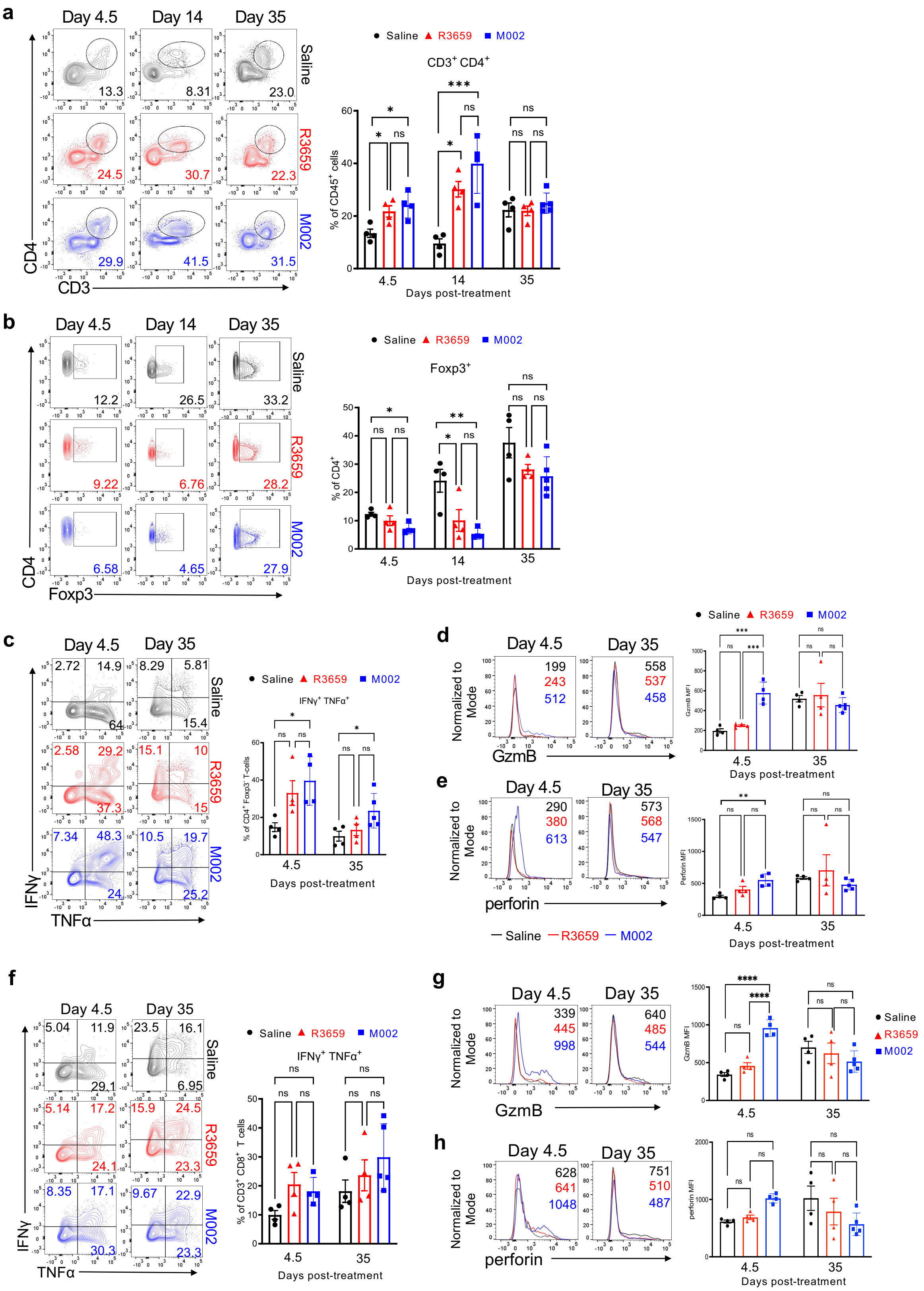

Supplementary Figure 4 (related to Figure 2): **The impact of IL-12 derived from M002 on T-cell response.**

Kinetic analysis of the abundance and function of $CD4^+$ T-cells (**a-e**) or the effector activity of $CD8^+$ T-cells (**f-h**) in brain tumors of mice implanted with GSC005 and treated with saline (black), R3659 (red) or M002 (blue), as in Fig. 1a. **a-c**) Representative plots (left), frequency (right) of $CD45^{hi}CD4^+CD3^+$ T-cells (**a**), $CD45^{hi}CD4^+CD3^+Foxp3^+$ Treg cells (**b**), and $IFN\gamma^+TNF\alpha^+CD45^{hi}CD4^+CD3^+Foxp3^-$ cells (**c**). **d-e**) Representative histograms (left) and MFI (right) of GzmB expression (**d**), and perforin expression (**e**) in $CD45^{hi}CD4^+CD3^+Foxp3^-$ cells. **f**) Representative plots (left), frequency (right) of $IFN\gamma^+TNF\alpha^+CD45^{hi}CD8^+CD3^+$ T-cells. **g,h**) Representative histograms (left) and MFI (right) of GzmB (**g**) and perforin (**h**) expression in $CD45^{hi}CD8^+CD3^+$ T-cells.

Each dot represents an individual mouse (n = 4 per group, except that n = 5 for day 35 in M002 group). ns, no significance, * $P < 0.05$, ** $P < 0.01$, *** $P < 0.001$ and **** $P < 0.0001$ (a-h, one-way ANOVA with Tukey's comparisons test at each time point). Bars, mean ± SEM. Source data (a-h) are provided in the Source Data file.

Supplementary Figure 5: related to Figure 3

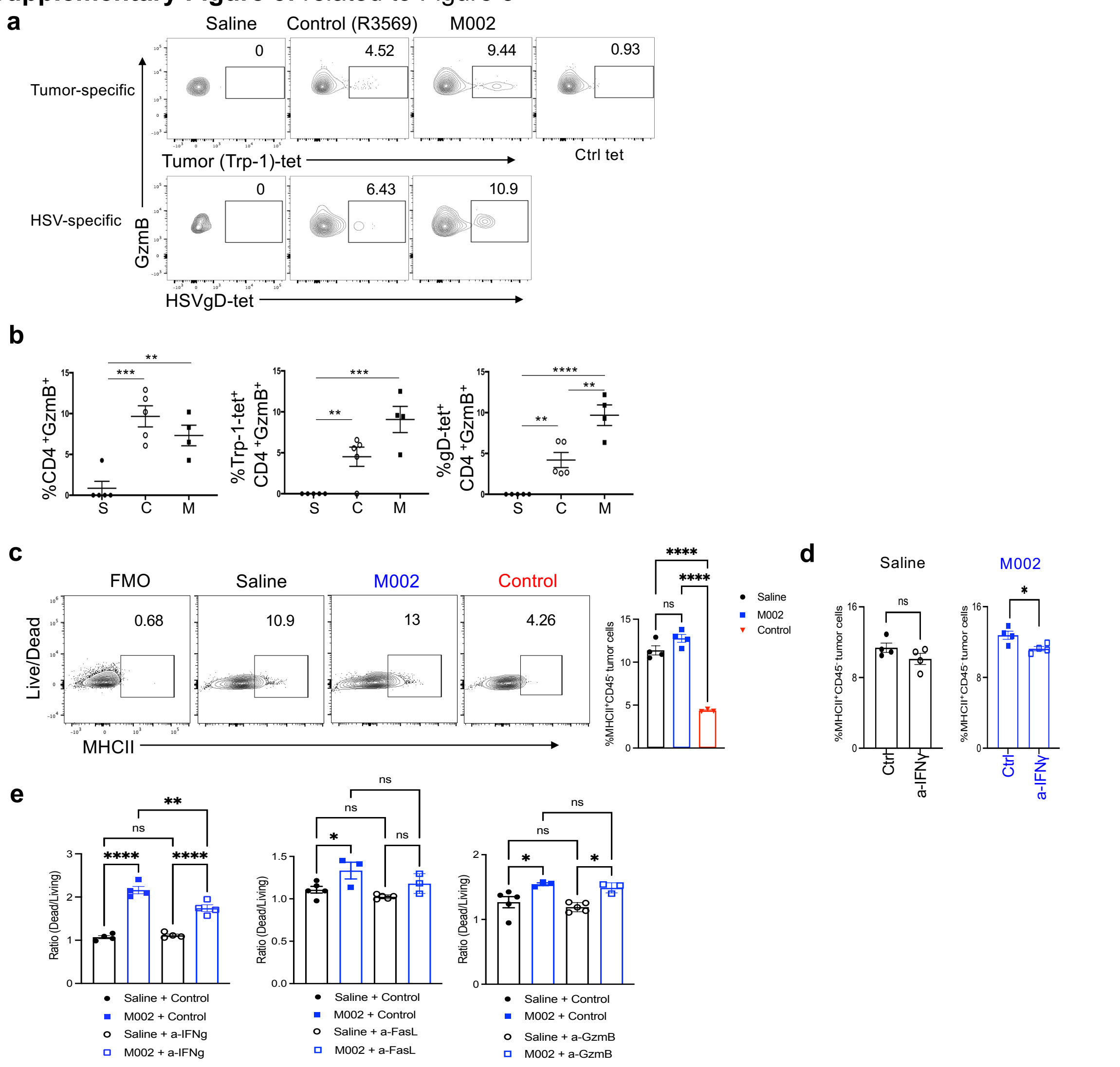

Supplementary Figure 5 (related to Figure 3): **Reprogrammed CD4⁺ T-cells eliminate tumor cells in an MHCII-dependent manner.**

a, b) B6 mice were intracranially injected with GL261-PVRL1 cells at day 0, and then injected with saline (S) (n = 5), control oHSV R3569 (C) (n = 5) or M002 (M) (n = 4) at day 10. $CD4^+$ T-cells from brain tumors were analyzed at day 7 post-treatment by flow cytometry. Representative plots (**a**) and frequency (**b**) of indicated tetramer (tet)-expressing $CD45^{hi}CD4^+CD3^+Foxp3^-GzmB^+$ cells. Ctrl tet, control I-Ab tetramer.

c, d) GSC005 tumor was established and treated as in Fig. 1a. $CD4^+$ T-cells were isolated and enriched from brain tumors (pooled from 4 mice per group) at day 14 post-treatment, and then co-cultured with GSC005 tumor cells at a 2:1 ratio in quadruplicates per group (except triplicates for the control group) for 16 hr before flow cytometry analysis (**c**). Cells without incubation with $CD4^+$ T-cells in triplicates were used as controls. Representative plots (left) and frequency (right) of $MHCII^+CD45^-$ live tumor cells. FMO, fluorescence minus one. **d**) $CD4^+$ T-cells were treated with anti-IFNγ or its isotype control antibody (Ctrl) for 1 hr prior to coculture with GSC005 cells in quadruplicates per group. Frequency of $MHCII^+CD45^-$ live tumor cells is shown.

e) $CD4^+$ T-cells were isolated and enriched from brain tumors (pooled from 4 mice per group) and then co-cultured with GSC005 tumor cells, as in **c**. $CD4^+$ T-cells were pre-incubated with anti-IFNγ, anti-FasL, anti-GzmB, or their respective isotype control antibody for 30 min (except 1 hr for anti-IFNγ) prior to co-culture in quadruplicates per group (anti-IFNγ), or in quintuples for the saline group and in triplicates for M002 group (anti-FasL and anti-GzmB). Quantification of specific killing ratios is shown.

ns, no significance, * $P < 0.05$, ** $P < 0.01$, *** $P < 0.001$ and **** $P < 0.0001$ (b ,d, unpaired two-tailed Student's t-test; c, e, one-way ANOVA with Tukey's comparisons test). Bars, mean ± SEM. Source data (b-e) are provided in the Source Data file.

Supplementary Figure 6: related to Figure 3

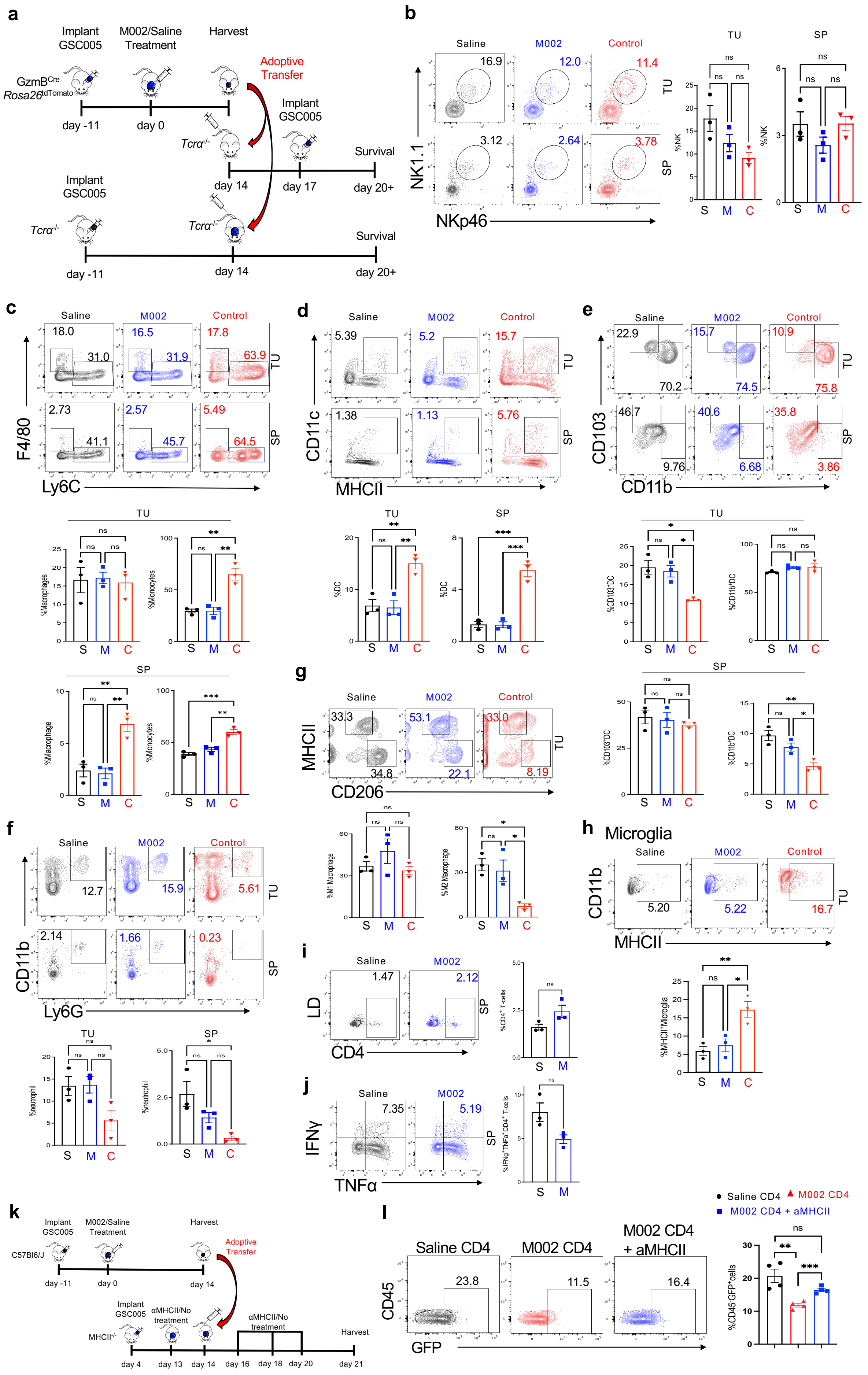

Supplementary Figure 6 (related to Figure 3): **Transfer of M002-programmed $CD4^+$ T-cells provides survival benefit.**

a) Schematic diagram of experiment for transfer of effector $CD4^+$ T-cells isolated from mice treated with M002 or saline, as described in Fig. 3e,f.

b-j) Immune cells from spleens or brain tumor of *Tcrα*$^{-/-}$ mice (n = 3 per group) in Fig. 3e at day 10 post-tumor implantation. Representative plots (left or upper) and frequency (right or bottom) of $CD45^{hi}NK1.1^+NKp46^+CD3^-$ NK cells (**b**), $CD45^{hi}CD11b^+Ly6G^-F4/80^+Ly6C^-$ macrophages or $CD45^{hi}CD11b^+Ly6G^-F4/80^-Ly6C^+$ monocytes (**c**), $CD45^{hi}CD11c^+MHCII^+$ DCs (**d**), $CD45^{hi}CD11c^+MHCII^+CD103^+CD11b^-$ DCs or $CD45^{hi}CD11c^+MHCII^+CD103^-CD11b^+$ DCs (**e**), $CD45^{hi}CD11b^+Ly6G^+$ neutrophils (**f**), $CD45^{hi}CD11b^+Ly6G^-F4/80^+Ly6C^-$ $MHCII^+CD206^-$ M1 macrophages or $CD45^{hi}CD11b^+Ly6G^-F4/80^+Ly6C^-$ $MHCII^-CD206^+$ M2 macrophages (**g**), $MHCII^+CD45^{med}CD11b^+$ microglia (**h**), $CD45^{hi}CD4^+CD3^+$ T-cells (**i**), and $IFN\gamma^+TNF\alpha^+CD45^{hi}CD4^+CD3^+$ T-cells (**j**).

k-l) The in vivo anti-tumor activity of $CD4^+$ T-cells required MHCII expression on tumor cells. **k**) Schematic diagram of experiment for $CD4^+$ T-cell transfer with or without the MHC blockade in vivo. At day -11, B6 mice were implanted with 50,000 GSC005 tumor cells. 11 days later mice were given either M002 or saline. At day 4 $MHCII^{-/-}$ mice (n = 4 per group) were implanted with 50,000 GSC005 tumor cells. At day 13, one set of $MHCII^{-/-}$ mice was given 200 μg of anti-MHCII antibody. At the following day (day 14), B6 mice treated with M002 or saline were harvested and 50,000 enriched $CD4^+$ T-cells were transferred into $MHCII^{-/-}$ mice bearing GSC005 tumor. The set of $MHCII^{-/-}$ mice that received anti-MHCII antibody were given another 3 doses of anti-MHCII every 2 days from day 16 to day 20. At day 21, brain tumor was harvested for analysis. **b**) Representative plots (left) and frequency (right) of live $CD45^-GFP^+$ tumor cells.

TU, tumor; SP, spleen. S, transfer of saline CD4; M, transfer of M002 CD4; C, *Tcrα*$^{-/-}$ mice with no transfer as controls. Data represent one of two independent experiments. ns, no significance, * $P < 0.05$, ** $P < 0.01$, and *** $P < 0.001$ (b-h, one-way ANOVA with Tukey's comparisons test; i-j, l, unpaired two-tailed Student's t-test). Bars, mean ± SEM. Source data (b-j, l) are provided in the Source Data file.

Supplementary Figure 7: related to Figure 4

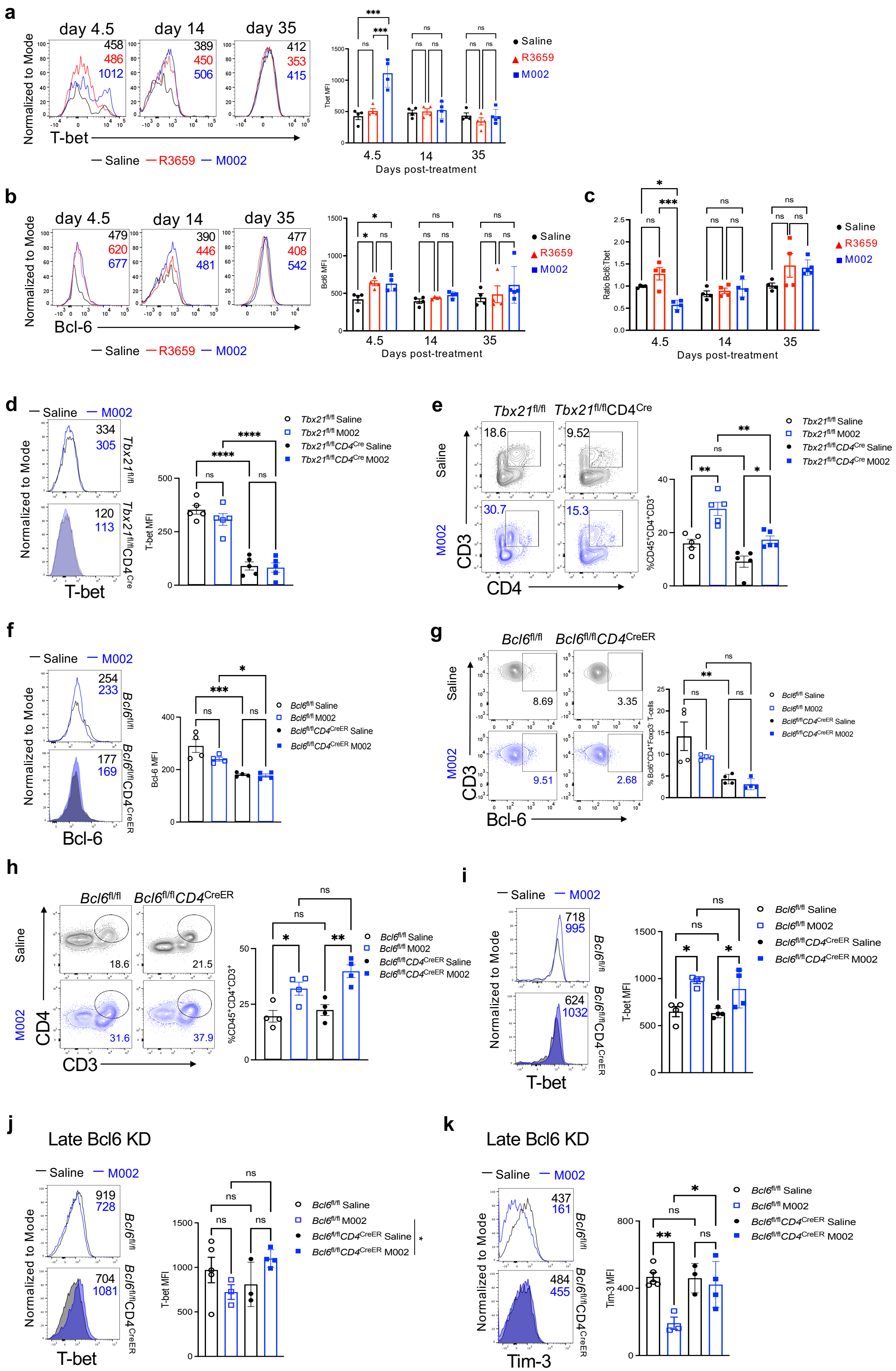

Supplementary Figure 7 (related to Figure 4): **The proper ratio of Bcl-6 to T-bet in $CD4^+$ T-cells navigates their enhanced anti-tumor capacity.**

a, **b**) Kinetic analysis of T-bet (**a**) or Bcl-6 (**b**) expression in $CD4^+$ T-cells in brain tumors of mice (n = 4 per group, except that n = 5 for day 35 in M002 group) implanted with GSC005 and treated with saline (black), R3659 (red) or M002 (blue), as in Fig. 1a. Representative histograms (left), and MFI (right) of T-bet (**a**) or Bcl-6 (**b**) expression in $CD45^{hi}CD4^+CD3^+Foxp3^-$ cells. **c**) The ratio of Bcl-6 to T-bet at indicated days as in a, b.
d, e) $CD4^+$ T-cells from brain tumors from Fig. 4d were analyzed at day 17 post-treatment by flow cytometry (n = 5 per group). Representative histograms (left), and MFI (right) of T-bet (**d**), and representative plots (left) and frequency (right) of $CD45^{hi}CD4^+CD3^+Foxp3^-$ cells (**e**).
f-i) $CD4^+$ T-cells from brain tumors from Fig. 4g were analyzed at day 12 post-treatment by flow cytometry (n = 4 per group). Representative histograms (left) and MFI (right) of Bcl-6 (**f**) or T-bet (**i**), and representative plots (left) and frequency (right) of $CD45^{hi}CD4^+CD3^+Foxp3^-Bcl\text{-}6^+$ cells (**g**) or $CD45^{hi}CD4^+CD3^+Foxp3^-$ cells (**h**).
j, k) $CD4^+$ T-cells from brain tumors from Fig. 4j were analyzed at day 46 post-treatment by flow cytometry. Representative histograms (left) and MFI (right) of T-bet (**j**) or Tim-3 (**k**) in $CD45^{hi}CD4^+CD3^+Foxp3^-$ cells. Saline $Bcl6^{fl/fl}$ (n = 5); M002 $Bcl6^{fl/fl}$ and Saline $Bcl6^{fl/fl}CD4^{CreER}$ (n = 3); M002 $Bcl6^{fl/fl}CD4^{CreER}$ (n = 4).

Data represent one of two independent experiments. Each dot represents an individual mouse. ns, no significance, * $P < 0.05$, ** $P < 0.01$, *** $P < 0.001$ and **** $P < 0.0001$ (a-k, one-way ANOVA with Tukey's comparisons test, a-c at each time point; unpaired two-tailed Student's t-test for statistical analysis noted in the legend of **j**). Bars, mean ± SEM. Source data (a-k) are provided in the Source Data file.

Supplementary Figure 8: related to Figure 5

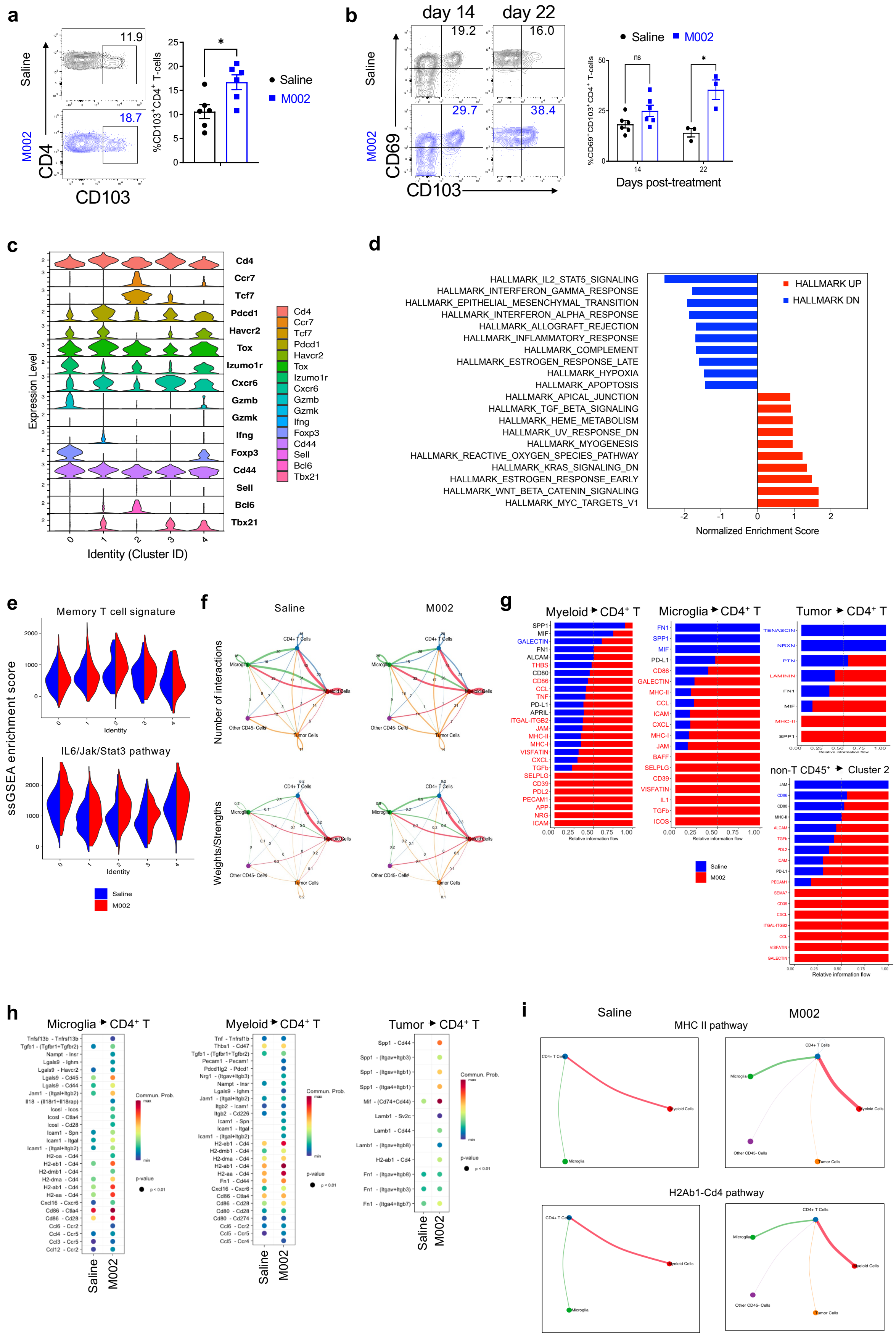

Supplementary Figure 8 *Cont'd*

j

NicheNet Analysis: Sender (Microglia, Myeloid, Tumor), Receiver (Total CD4)
Gene upregulated in M002 vs Saline

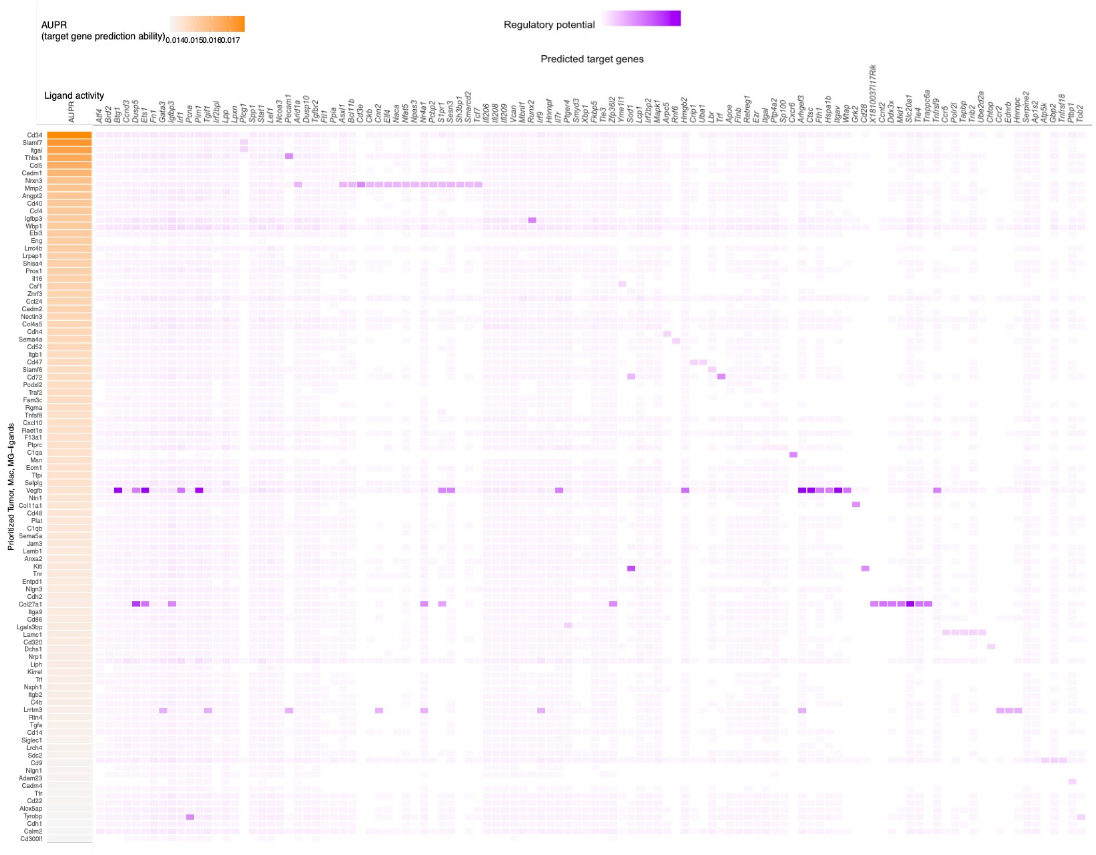

Supplementary Figure 8 (related to Figure 5): **The tumor-associated MHCII pathway facilitates the formation of a memory CD4$^+$ T-cell subset.**

a) CD103 expression in CD4$^+$ T-cells in brain tumors of mice (n = 6 per group) implanted with GSC005 and treated with saline or M002, as in Fig. 1a, at day 35 post-treatment. Representative plots (left) and frequency (right) of CD103$^+$CD45^{hi}CD4$^+$CD3$^+$Foxp3$^-$ cells.
b) Mice were implanted with GL261-PVRL1 and treated with saline or M002 10 days later. Analysis of CD4$^+$ T-cells in brain tumors at days 14 (n = 6 per group) and 22 (n = 3 per group) post-treatment. Representative plots (left) and frequency (right) of CD69$^+$CD103$^+$CD45^{hi}CD4$^+$CD3$^+$Foxp3$^-$ cells. ns, no significance and * $P < 0.05$ (a-b, unpaired two-tailed Student's t-test). Bars, mean ± SEM. Source data (a-b) are provided in the Source Data file.

c) Violin plots showing the expression of marker genes across clusters, as in Fig. 5a; the y-axis represents the normalized gene expression value.
d) Bar chart of top 10 upregulated and top 10 downregulated Hallmark pathways in cluster 2 versus all the other clusters in Fig. 5a-d.
e) Comparison of ssGSEA scores of T-cell memory signature and IL6/Jak/Stat3 pathway across clusters in M002-treated CD4$^+$ T-cells compared to saline-treated controls.
f-i) CellChat analysis of cell-cell interactions with total CD4$^+$ T-cells or cluster 2 cells, as in Fig. 5g-j.
j) Predictions from NicheNet denote target genes in CD4$^+$ T-cells (receivers) in the M002-treated group as compared to saline from microglia, myeloid and tumor cells (senders). The ligands are ranked based on their activity scores (orange color) while their regulation potential on target genes is colored in violet.

Supplementary Figure 9: related to Figure 5

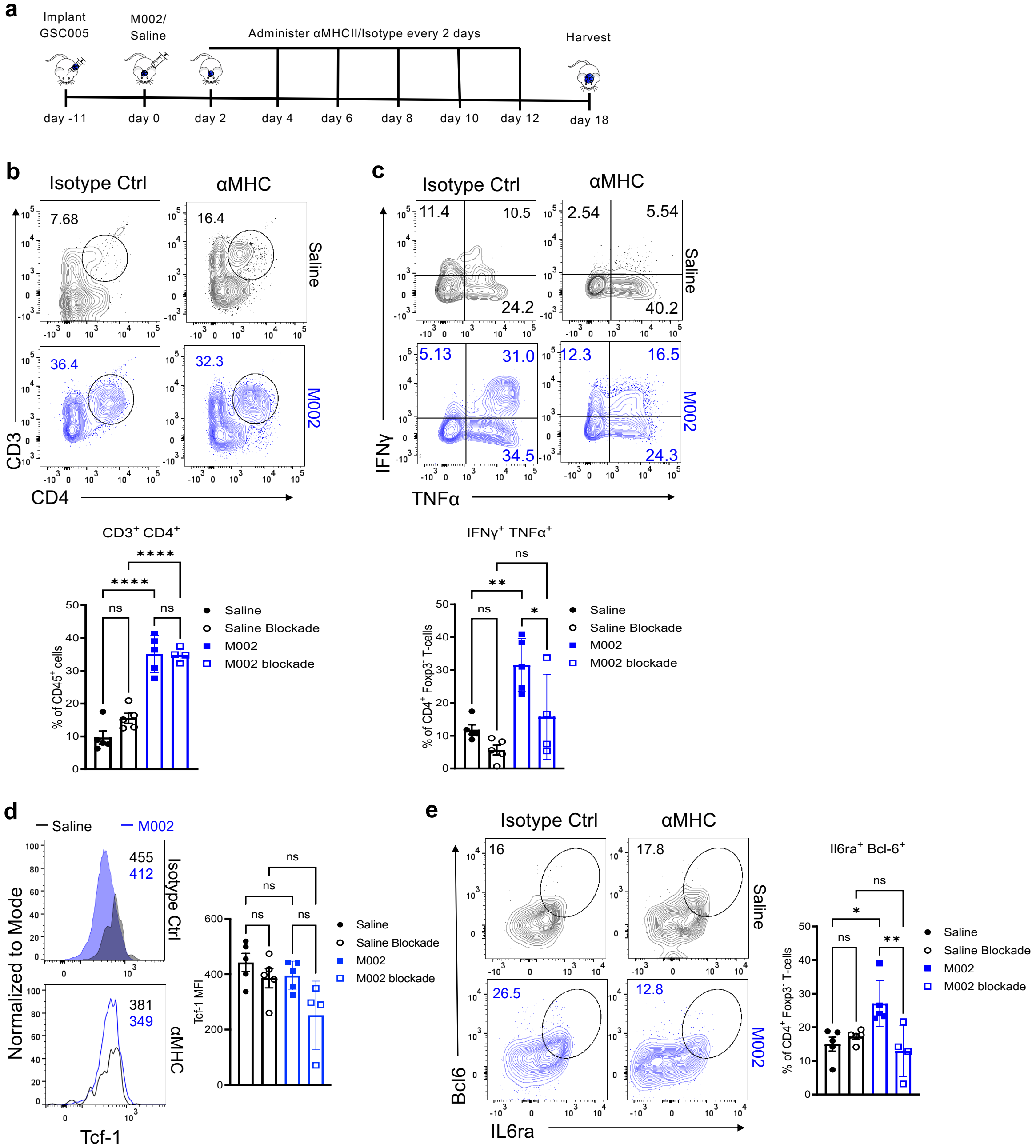

Supplementary Figure 9 (related to Figure 5): **The impact of MHCII blockade on CD4⁺ T-cell response in vivo.**

a) Schematic diagram of experiment for MHCII blockade *in vivo*. B6 mice were implanted with GSC005 at day -11 and then treated with saline or M002 at day 0, as in Fig. 1a. Mice were given a total of 6 doses of either anti-MHCII or isotype control antibody starting 2 days post-treatment. Five days (at day 18) after the final dose of antibody brain tumors were harvested for flow analysis of CD4⁺ T-cells for their abundance (**b**), effector activity (**c**) and potential memory phenotype (**d, e**). **b, c, e**) Representative plots (upper or left), frequency (bottom or right) of $CD45^{hi}CD4^{+}CD3^{+}$ T-cells (**b**), $IFN\gamma^{+}TNF\alpha^{+}CD45^{hi}CD4^{+}CD3^{+}Foxp3^{-}$ cells (**c**), and $Bcl6^{+}IL6ra^{+}CD45^{hi}CD4^{+}CD3^{+}Foxp3^{-}$ (**e**). **d**) Representative histograms (left) and MFI (right) of Tcf-1 expression in $CD45^{hi}CD4^{+}CD3^{+}Foxp3^{-}$ T-cells. Each dot represents an individual mouse (n = 5 per group, except that n = 4 for M002 Blockade group). ns, no significance, * $P < 0.05$, ** $P < 0.01$ and **** $P < 0.0001$ (b-e, one-way ANOVA with Tukey's comparisons test). Bars, mean ± SEM. Source data (b-e) are provided in the Source Data file.

Supplementary Figure 10: related to Figure 6

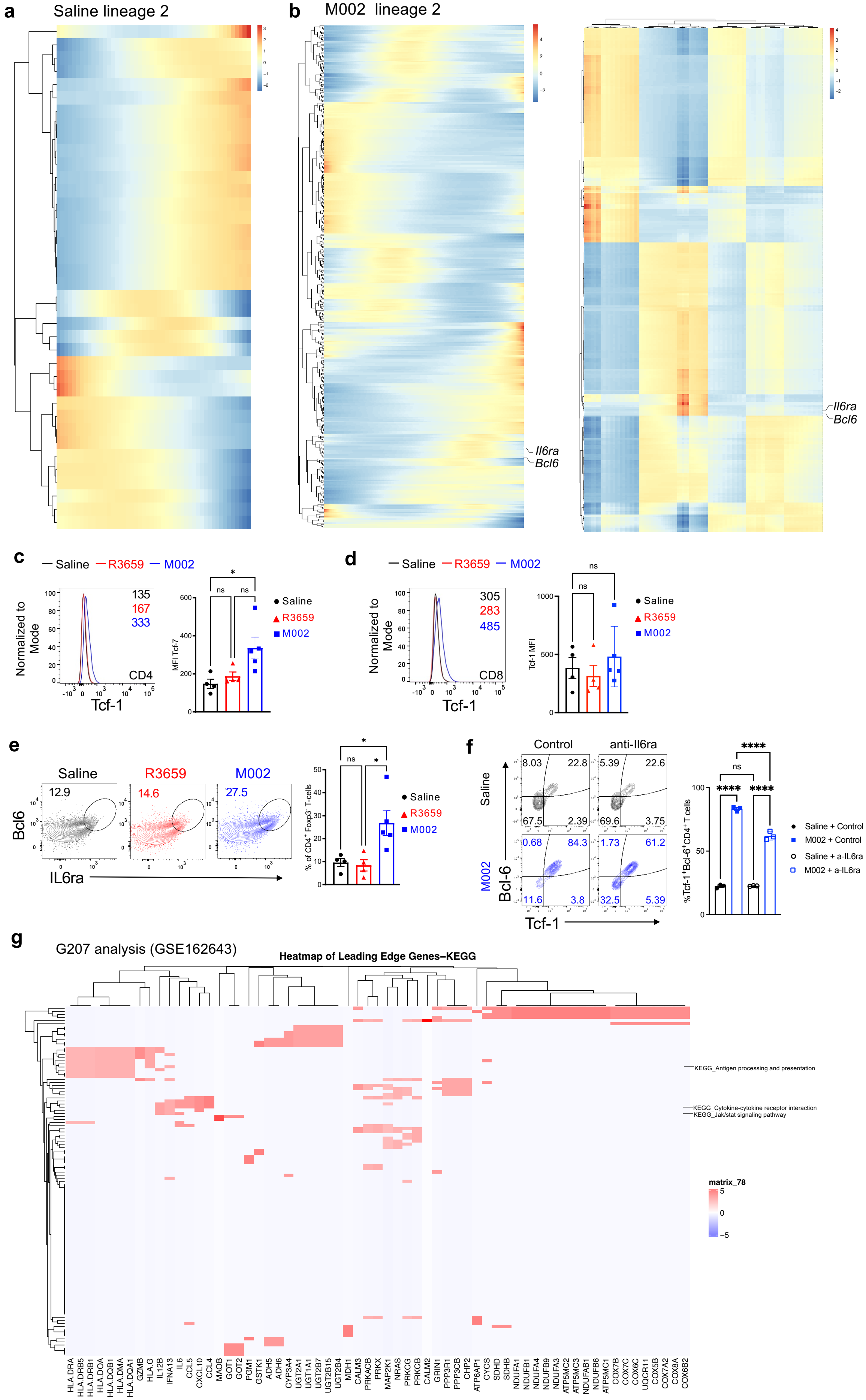

Supplementary Figure 10 (related to Figure 6): **The reciprocal IL6ra-Bcl-6 regulatory axis regulates the memory $CD4^+$ T-cell differentiation.**

a, b) Heatmaps show differentially regulated genes (DRGs) along the lineage 2 pseudotime for saline (**a**) and M002 (**b**, left) treated groups. **b**, right) Heatmap shows clustered pseudotime DRGs in lineage 2 trajectory in the M002-treated group. The color scale indicates log-expression values of DRGs. The co-regulation of *Bcl6* and *Il6ra* is indicated.

c-e) Analysis of $CD4^+$ T-cells (**c, e**) or $CD8^+$ T-cells (**d**) in brain tumors of mice (n = 4 per group, except that n = 5 for M002 group) implanted with GSC005 and treated with saline (black), R3659 (red) or M002 (blue), as in Fig. 1a, at day 35 post-treatment. Representative histograms (left) and MFI (right) of Tcf-1 expression in $CD45^{hi}CD4^+CD3^+Foxp3^-$ cells (**c**) or $CD45^{hi}CD8^+CD3^+$ cells (**d**). Representative plots (left), and frequency (right) of $Bcl6^+IL6ra^+CD45^{hi}CD4^+CD3^+Foxp3^-$ cells (**e**).

f) Flow cytometry plots (left) and frequency (right) of $Tcf\text{-}1^+Bcl\text{-}6^+CD4^+CD3^+Foxp3^-$ T-cells in the co-culture of $CD4^+$ T-cells and irradiated splenocytes pulsed with Trp-1 in the presence of anti-IL6ra or its isotype control antibody in triplicates per group, as in Fig. 6f-h.

ns, no significance, * $P < 0.05$ and **** $P < 0.0001$ (c-f, one-way ANOVA with Tukey's comparisons test). Bars, mean ± SEM. Source data (c-f) are provided in the Source Data file.

g) Heatmap shows the leading edge genes revealed in the KEGG pathways (see Supplementary Data 4) significantly upregulated in GBM patients post-G207 therapy compared to pre-treatment with survival as a covariate. Antigen processing and presentation, and IL6-related pathways are noted.

Supplementary Figure 11

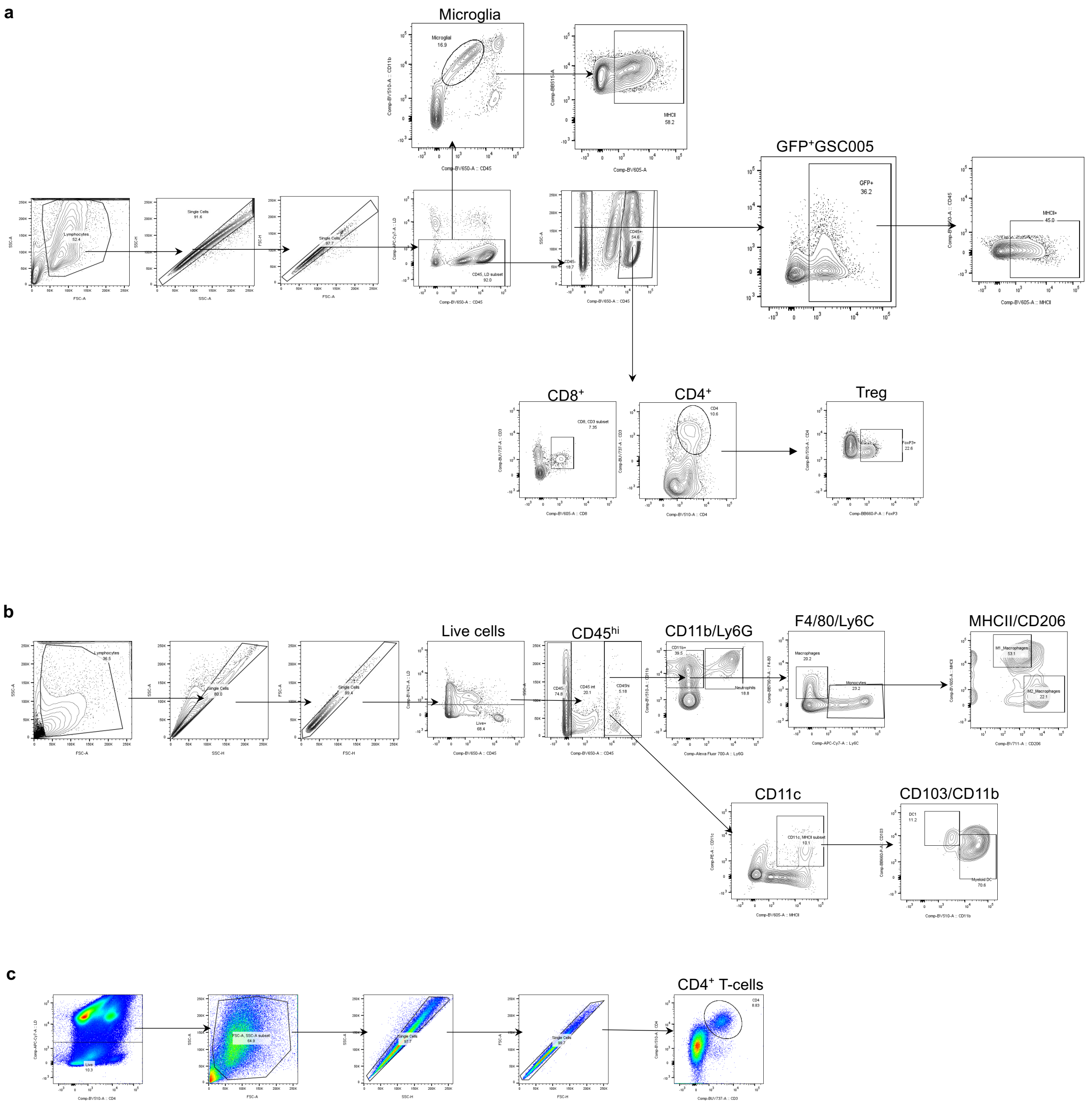

Supplementary Figure 11: Gating Strategy.

a) Strategy for analysis of microglia, T cells and tumor cells.

b) Strategy for analysis of innate immune cells.

c) Strategy for analysis of $CD4^+$ T-cells in the in vitro co-culture assays.
