# Supplementary material for: Oncolytic reprogramming of tumor microenvironment shapes CD4 T-cell memory via the IL6ra-Bcl6 axis for targeted control of glioblastoma
Source: Nat Commun. 2025 Jan 30;16:1095. doi: 10.1038/s41467-024-55455-9 (PMC11782536; doi:10.1038/s41467-024-55455-9)
Supplement: Supplementary file 2 — Description of Additional Supplementary Files [file 41467_2024_55455_MOESM2_ESM.pdf]

Description of Additional Supplementary Files

Supplementary Data 1: List of significantly differentially expressed genes in cluster 2 CD4+ T-cells as compared to all other clusters as shown in Fig. 5d. Highlighted genes in bold indicate the top 10 ranked genes (based on log2 fold change (FC) and adjusted P values) used for TCGA-GBM survival analysis in Fig. 6j. Two-sided non-parametric Wilcoxon Rank Sum Test with FDR adjustments for multiple comparisons.

Supplementary Data 2: Differentially regulated genes (DRGs) along lineage 2 pseudotime for saline and M002 treated groups (from top to bottom), as shown in Supplementary Fig. 10a, b.

Supplementary Data 3: Genes coregulated with Bcl6 based on pseudotime for lineage 2 in M002-treated CD4+ T-cells, related to Fig. 6d. P-values (two-sided Wald tests) indicate the significance of the association with multiple testing correction (FDR). Metrics include adjusted p-values (FDR-corrected) and test statistics for each gene.

Supplementary Data 4: Leading edge analysis of G207 dataset (from top to bottom matching the heatmap in Supplementary Fig. 10g).

Supplementary Data 5: CIBERSORT Analysis of tumor specimens from G207 trial, related to Fig. 6i. P-values indicate the significance of deconvolution results derived from one-sided permutation testing to assess the enrichment of cell types against a null model. Deconvolution scores represent relative or absolute cell-type proportions inferred using linear support vector regression (SVR). Root Mean Squared Error (RMSE) quantifies the fit of predicted gene expression values to observed data. Correlation coefficients reflect the strength of the linear relationship between predicted and observed gene expression profiles,

where higher values indicate stronger model performance. Multiple comparisons are not inherently adjusted.

Supplementary Data 6: List of reagents and resources used in this manuscript.

All are cited as Supplementary Data 1-6 in the text.
