# Supplementary material for: Oncolytic reprogramming of tumor microenvironment shapes CD4 T-cell memory via the IL6ra-Bcl6 axis for targeted control of glioblastoma
Source: Nat Commun. 2025 Jan 30;16:1095. doi: 10.1038/s41467-024-55455-9 (PMC11782536; doi:10.1038/s41467-024-55455-9)
Supplement: Supplementary file 9 — Reporting Summary [file 41467_2024_55455_MOESM9_ESM.pdf]

nature portfolio

Corresponding author(s): Jianmei Leavenworth

Last updated by author(s): Nov 29, 2024

Reporting Summary

Nature Portfolio wishes to improve the reproducibility of the work that we publish. This form provides structure for consistency and transparency in reporting. For further information on Nature Portfolio policies, see our Editorial Policies and the Editorial Policy Checklist.

Statistics

For all statistical analyses, confirm that the following items are present in the figure legend, table legend, main text, or Methods section.

n/a	Confirmed	
☐	☒	The exact sample size (*n*) for each experimental group/condition, given as a discrete number and unit of measurement
☐	☒	A statement on whether measurements were taken from distinct samples or whether the same sample was measured repeatedly
☐	☒	The statistical test(s) used AND whether they are one- or two-sided<br>*Only common tests should be described solely by name; describe more complex techniques in the Methods section.*
☒	☐	A description of all covariates tested
☐	☒	A description of any assumptions or corrections, such as tests of normality and adjustment for multiple comparisons
☐	☒	A full description of the statistical parameters including central tendency (e.g. means) or other basic estimates (e.g. regression coefficient) AND variation (e.g. standard deviation) or associated estimates of uncertainty (e.g. confidence intervals)
☐	☒	For null hypothesis testing, the test statistic (e.g. *F*, *t*, *r*) with confidence intervals, effect sizes, degrees of freedom and *P* value noted<br>*Give P values as exact values whenever suitable.*
☒	☐	For Bayesian analysis, information on the choice of priors and Markov chain Monte Carlo settings
☒	☐	For hierarchical and complex designs, identification of the appropriate level for tests and full reporting of outcomes
☐	☒	Estimates of effect sizes (e.g. Cohen's *d*, Pearson's *r*), indicating how they were calculated

Our web collection on statistics for biologists contains articles on many of the points above.

Software and code

Policy information about availability of computer code

Data collection	TCGA-GBM dataset was obtained using the cSurvival portal. The G207 RNAseq data were extracted from the NCBI GEO under accession number GSE162643. Flow cytometry data was collected using BD FacsDiva software version 8.0.
Data analysis	The following software or tools were used for RNAseq analysis: GREIN interactive web pipeline; GSEA; CIBERSORTx algorithm<br>The following software or tools were used for scRNAseq analysis: Cell Ranger Count v7.1.0, 10× Genomics; DoubletFinder package (version 2.0.4); R Studio (version 2023.09.0+463, R version 4.3.3); Seurat package (v5); RunPCA function; clustree package; RunUMAP function; FindMarkers function; dittoseq package; EnhancedVolcano package; clusterProfiler; fgsea and escape packages; scCustomize; GSEAVis; scVelo (v0.2.5, python package); Slingshot; tradeSeq package; CellChat package (v2.0); NicheNet package<br>Flow cytometry data were analyzed using FlowJo software version 10.6.<br>GraphPad Prism V9.5 was used for statistical analysis.

For manuscripts utilizing custom algorithms or software that are central to the research but not yet described in published literature, software must be made available to editors and reviewers. We strongly encourage code deposition in a community repository (e.g. GitHub). See the Nature Portfolio guidelines for submitting code & software for further information.

Data

Policy information about availability of data

All manuscripts must include a data availability statement. This statement should provide the following information, where applicable:

- Accession codes, unique identifiers, or web links for publicly available datasets
- A description of any restrictions on data availability
- For clinical datasets or third party data, please ensure that the statement adheres to our policy

The single-cell RNAseq data are deposited in the NCBI GEO under accession number GSE246895. All data generated or analyzed during this study are included in this article and its supplementary information. Source data are provided with this paper.

Research involving human participants, their data, or biological material

Policy information about studies with human participants or human data. See also policy information about sex, gender (identity/presentation), and sexual orientation and race, ethnicity and racism.

Reporting on sex and gender	No human participants have been enrolled in this study. The findings of this study, extrapolated and tested on availabe datasets, including our G207 clinical trial, cited accordingly, have no sex or gender biases.
Reporting on race, ethnicity, or other socially relevant groupings	N/A
Population characteristics	N/A
Recruitment	N/A
Ethics oversight	N/A

Note that full information on the approval of the study protocol must also be provided in the manuscript.

Field-specific reporting

Please select the one below that is the best fit for your research. If you are not sure, read the appropriate sections before making your selection.

☒ Life sciences ☐ Behavioural & social sciences ☐ Ecological, evolutionary & environmental sciences

For a reference copy of the document with all sections, see nature.com/documents/nr-reporting-summary-flat.pdf

Life sciences study design

All studies must disclose on these points even when the disclosure is negative.

Sample size	Sample size was not specifically predetermined, but the number of mice used was consistent with previous experience with similar experiments. The precise number of animals used is provided in the figure legend.
Data exclusions	No exclusion of data points was used.
Replication	Experiments were repeated independently or performed with biological replicates. The precise number of repeats is provided in the figure legend.
Randomization	In general, all mice were obtained from the same cohort, and were randomly assigned to groups for injection with tumor cells. For immune memory studies, age-matched naive mice were included as controls.
Blinding	Experiments were performed in an unblinded fashion (included in the Methods section), as blinding was not applicable for this study. The data collection and analyses rely on objective measures.

Reporting for specific materials, systems and methods

We require information from authors about some types of materials, experimental systems and methods used in many studies. Here, indicate whether each material, system or method listed is relevant to your study. If you are not sure if a list item applies to your research, read the appropriate section before selecting a response.

Materials & experimental systems

n/a	Involved in the study	
☐	☒	Antibodies
☐	☒	Eukaryotic cell lines
☒	☐	Palaeontology and archaeology
☐	☒	Animals and other organisms
☒	☐	Clinical data
☒	☐	Dual use research of concern
☒	☐	Plants

Methods

n/a	Involved in the study	
☒	☐	ChIP-seq
☐	☒	Flow cytometry
☒	☐	MRI-based neuroimaging

Antibodies

Antibodies used	All antibodies including clone information, concentration, catalogue numbers and company source are provided in the Methods section.
Validation	All antibodies used for flow cytometry are commercially available and validation materials are available on the appropriate websites from the vendors.

Eukaryotic cell lines

Policy information about cell lines and Sex and Gender in Research

Cell line source(s)	Murine GSC005 provided by Dr. Inder M. Verma at the Salk Institute for Biological Studies; Murine GL261 cells obtained from the Division of Cancer Treatment Tumor Repository; Vero cells obtained from the American Type Culture Collection (CCL-81)
Authentication	Results from these cell lines have been published. The references are included in the Methods section.
Mycoplasma contamination	All the cell lines used were confirmed pathogens-free, including mycoplasma-free, by Charles River Research Animal Diagnostic Services.
Commonly misidentified lines (See ICLAC register)	N/A

Animals and other research organisms

Policy information about studies involving animals; ARRIVE guidelines recommended for reporting animal research, and Sex and Gender in Research

Laboratory animals	All mice used on C57BL/6J background were 6 to 11 weeks of age unless otherwise specified
Wild animals	N/A
Reporting on sex	Both sexes (males or females) were randomly included for comparison groups in all experiments in an unblinded fashion
Field-collected samples	N/A
Ethics oversight	All animal experiments were performed in compliance with federal laws and institutional guidelines as approved by the Institutional Animal Care and Use Committee (IACUC) at the University of Alabama at Birmingham (UAB).

Note that full information on the approval of the study protocol must also be provided in the manuscript.

Plants

Seed stocks	N/A
Novel plant genotypes	N/A
Authentication	N/A

Flow Cytometry

Plots

Confirm that:

- [x] The axis labels state the marker and fluorochrome used (e.g. CD4-FITC).
- [x] The axis scales are clearly visible. Include numbers along axes only for bottom left plot of group (a 'group' is an analysis of identical markers).
- [x] All plots are contour plots with outliers or pseudocolor plots.
- [x] A numerical value for number of cells or percentage (with statistics) is provided.

Methodology

Sample preparation	The procedures for the isolation and staining cells for flow cytometry analysis as well as intracellular stains are provided in the Methods section.
Instrument	BD LSRII or FACSymphony for analysis and BD FACSAria for sorting
Software	Flow cytometry data was collected using BD FacsDiva software version 8.0. Data were analyzed using FlowJo software version 10.6.
Cell population abundance	After sorting, sorted cells were re-run with the exact setting on the same instrument. Sorted cells with a purity >90% were subject to the following experiments.
Gating strategy	Forward versus side scatter (FSC vs SSC) gating was used to identify cells of interest and exclude debris and dead cells. The fixable viability dye was included in the staining and used to exclude dead cells. A forward scatter height (SSC-H) vs. forward scatter area (SSC-A) density plot was used to exclude doublets.

- [x] Tick this box to confirm that a figure exemplifying the gating strategy is provided in the Supplementary Information.
